# Developing a patient-driven chronic obstructive pulmonary disease (COPD) research agenda in the U.S.

**DOI:** 10.1186/s41687-021-00399-7

**Published:** 2021-12-04

**Authors:** Inga Gruß, Gretchen M. McCreary, Ilya Ivlev, Mary Ellen Houlihan, Barbara P. Yawn, Cara Pasquale, William Clark, Richard A. Mularski

**Affiliations:** 1 Kaiser Permanente Center for Health Research, 3800 N. Interstate Ave, Portland, OR 97227 USA; 2 COPD Foundation, 3300 Ponce de Leon Blvd, Miami, FL 33134 USA; 3 COPD Patient-Powered Research Network Governing Board, Washington, DC USA; 4 University of Minnesota, 6500 Delaware, Minneapolis, MN 55449 USA

**Keywords:** Chronic obstructive pulmonary disease, Patient-generated research topics, Research agenda, Patient and public involvement, Open-ended survey responses

## Abstract

**Background:**

To document a generalizable process for developing a patient-prioritized chronic obstructive pulmonary disease (COPD) research agenda and to provide an overview of domains that were developed in response to people living with COPD and caregivers’ suggestions for research.

**Methods:**

Adults with COPD and caregivers who are members of the COPD Patient-Powered Research Network (PPRN) provided suggestions for COPD-related research through a self-administered, online survey. These responses were analyzed with a content analysis approach: domains for categorizing all survey responses were created, then all responses were categorized independently by a group of researchers, then these categorizations were adjudicated, and finally a density map was created that represented the number of responses in each of the domains.

**Results:**

At the time of analysis, 6157 adults had fully completed the baseline survey. Survey responses were categorized across seven domains as follows: 22.5% of all responses fell into the domain family/social/community research, 20.8% of all responses fell into the domain well-being, 15% of all responses fell into the domain curative research, 14.6% of all responses fell into the domain biomedical therapies, 10.5% of all responses fell into the domain policy concerns, 6% of all responses fell into the domain holistic therapies and 10.7% of all responses fell into the domain ambiguous comments that could not be translated into concrete research topics.

**Conclusion:**

Using qualitative open-ended survey responses from the COPD PPRN registrants, we were able to identify six key domains of research about COPD that are considered most important by patients. These domains differ in content from prior scientist-led efforts to develop priorities for COPD research, demonstrating the ongoing importance of involving patients and their caregivers in determining research priorities. The results suggest the field can more closely align research efforts to patient priorities by considering the identified domains.

## Plain English summary

The COPD Patient-Powered Research Network (PPRN) asked adults with COPD and their caregivers to suggest areas for future research on COPD. Researchers analyzed 7336 survey responses and created six research areas to reflect their suggestions: (1) family/social/community research, (2) well-being, (3) research on a cure for COPD, (4) new drugs and treatments, (5) policy concerns, and (6) natural therapies. Several research topics patients and their caregivers identified as important have received little attention from researchers in the past and could be valuable topics for researchers to explore.

## Background

More than 16 million individuals in the U.S. are diagnosed with chronic obstructive pulmonary disease (COPD). COPD is the fourth leading cause of death in the U.S., and resulted in health care expenditures of $36 billion in 2010 (projected to rise to $49 billion in 2020) [1]. However, research on COPD is relatively limited; according to the COPD Foundation, there are 780 ongoing clinical trials related to COPD, compared with 41,000 trials related to cancer [2]. Promoting COPD research is important to achieve better health outcomes for COPD patients [3]. However, to ensure the importance and relevance of research topics to those affected by the disease, it is critical to give patients and their families a voice in shaping an expanded COPD research agenda [4].

Engaging patients continuously across the research spectrum advances patient-centered outcomes [5, 6]. Engaging stakeholders can also establish public trust [7], allow for development of treatment and interventions that reflect stakeholder priorities [8], improve professional capacity and competence among stakeholder groups [9], improve health-related outcomes [10], and decrease health inequalities [11]. Despite the recognition that stakeholder engagement in COPD research is important [12] and feasible [13], to date, stakeholder engagement in COPD research has been limited and has only taken place on small scales [14, 15]. For example, in one study stakeholder groups such as clinicians, researchers and health care representatives were asked to rank the importance of research topics, but no stakeholders were involved in generating the topics [16]. In another study that identified COPD-related research priorities, involved stakeholders were limited to representatives of professional and research organizations; only a few represented patient advocacy groups [17].

To our knowledge, there are no patient-driven COPD-related research agendas. Creating an agenda of this sort is important for ensuring the relevance of COPD research to patients and their caregivers. In this manuscript, we document a generalizable process for developing a patient-prioritized COPD research agenda and provide an overview of domains and topics that reflect suggestions for future research made by people living with COPD.

## Methods

### Study setting and overview

This study was a collaboration between the COPD Foundation—an advocacy group for patients with COPD and their caregivers—and the Kaiser Permanente Center for Health Research. The COPD Foundation hosts the COPD patient-powered research network (PPRN), a U.S.-wide network of over 8000 people with COPD and at risk for COPD who have agreed to share their health information for research purposes. The network is operated and governed by people with COPD and their partners. IRB statement here that was withheld for double-blind peer review. The Kaiser Permanente Northwest Institutional Review Board determined that this project was exempted from human subject’s research review and it was approved by the COPD Foundation IRB of record.

### Participants

The COPD PPRN recruited participants digitally through COPD360social—a free online social network focused on COPD for anyone affected by COPD—and Facebook, as well as through community outreach conducted by the COPD PPRN governing board members and COPD Foundation state captains. Any English-speaking person over the age of 18 who meets at least one of the following criteria is eligible to join the COPD PPRN:

- has been told by a doctor that they have COPD,
- has a family history of respiratory disease,
- is a current or former smoker,
- is symptomatic of respiratory disease (including but not limited to coughing, shortness of breath, or wheezing),
- has had a possible or known environmental/occupational exposure that may put them at risk for a respiratory disease (including but not limited to second-hand smoke and indoor and/or outdoor air pollution),
- is the caregiver of a person with COPD.

Individuals can join by creating an online account and completing online informed consent.

### Data collection

**Fig. 1:**
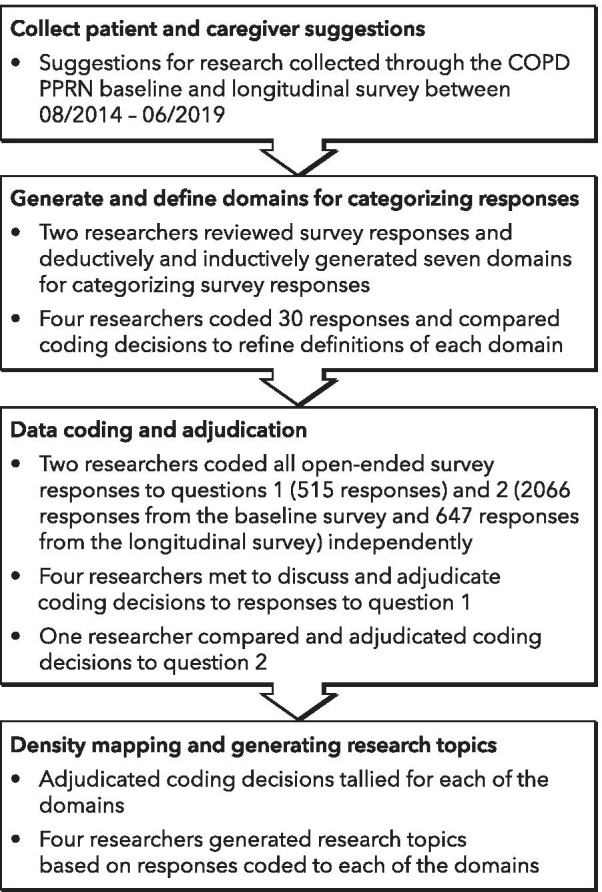
Data collection and analysis process

At the time of registration on the website, each future COPD PPRN member is asked to fill out a baseline survey, which includes questions about basic demographics, existing medical conditions, current COPD symptoms, and interest in research. The survey takes about 20 min to complete. Each member is also invited to complete an annual longitudinal survey beginning one year after enrollment that asks questions about updates to contact information, medical history, COPD symptoms, interest in research, and any other comments. Two open-ended survey questions captured data relevant for this study: 1) “What research in COPD matters most to you?” and 2) “What other things do you want to tell us about research for COPD that matters to you, or do you have any other comments for the researchers?” Question one was asked in the baseline survey, question two was asked both in the baseline and longitudinal survey. Question one includes five set responses (looking at how different medications can help my breathing, looking at ways to help cure my disease, looking at ways to help me live longer, looking at ways to keep me comfortable even if not living longer, and looking at ways my spouse/partner/caregiver can help me) that respondents can select from, checking all that apply, and an open-ended “other” field where they can type in free text. Question two provided a free text field for responses. Responses to these questions were not mandatory for completion of the survey, and many respondents left them blank. Figure 1 illustrates the data collection and analysis process. Data for this study was collected through the baseline survey between August 2014 and June 2019 and the longitudinal survey between July 2016 and June 2019.

### Data analysis

#### Generating and defining domains

A COPD expert (RM) and a qualitative research expert (IG) independently reviewed all open-ended responses to question one. The qualitative research expert deductively generated overarching domains to categorize these responses; the COPD expert combined deductive and inductive strategies based on prior research to generate new domains [17]. They then independently coded 30 items into the seven resulting domains. After comparing and discussing their respective coding decisions, they revised the definitions of each of the domains. Responses to question two were then assessed; no revisions were made to the existing domains as they sufficiently captured these responses.

#### Data coding and adjudication

The coding dictionary was shared with two additional researchers (GM, II) who independently coded a second set of 30 training responses. The researchers met and compared the coding decisions and resolved disagreements through discussion. GM and II then independently coded all open-ended responses to questions 1 and 2. Responses could be categorized into up to three domains, accounting for multiple ideas within a free-text response as well as the conceptual overlap between domains. For example, the comment “weight loss, depression, aggression” was categorized as relevant to the domains well-being and holistic therapies. “Full agreement” was defined as identical coding decisions among researchers, “ partial agreement” was defined as one divergent and one common coding decision, and “disagreement” was defined as no overlapping coding decisions among the researchers.

All four researchers met to discuss and adjudicate the coding decisions to question 1. When there were coding disagreements, GM and II explained their respective coding decisions and consensus categorization was achieved among all four researchers through discussion. We adopted a different approach for adjudicating coding decisions for question 2 as a lot of responses fell into domain 7, and discussing all responses with four researchers was time intensive. Therefore, coding disagreements for question two were reviewed only by IG, who resolved coding disagreements in line with coding dictionary definitions.

#### Density mapping and generating research topics

Next, a density map that illustrated the concentration of responses in the different domains was created (see Table 1). The purpose of the density map was to understand how many participant responses had been categorized to each domain. Research topics were then developed through discussion among the core coding team and then shared with the larger research team for feedback. As the goal was to turn participant responses into topics that could be researched, the number of research topics generated for each domain reflected the number of participant responses categorized to each domain. The researchers created more research topics for domains with more categorized responses than those with fewer categorized responses.

**Table 1:** Coding agreement and density mapping

	What research in COPD matters most to you? (Baseline survey)	What other things do you want to tell us about research for COPD that matters to you, or do you have any other comments for the researchers? (Baseline survey)	What other things do you want to tell us about research for COPD that matters to you, or do you have any other comments for the researchers? (Longitudinal survey)
Number of responses	n = 515 (%)	n = 2066 (%)	n = 647 (%)
*Coding agreement before adjudication*			
Full coding agreement	339 (66)	1100 (53.2)	342 (52.8)
Partial agreement	98 (19)	112 (5.4)	14 (2.1)
Disagreement	76 (15)	855 (41.3)	291 (44.9)
*Density mapping to each domain after adjudication*			
Domain 1 (family/social/community research)	116 (22.5)	104 (5)	16 (2.4)
Domain 2 (well-being)	107 (20.8)	142 (6.8)	12 (1.8)
Domain 3 (curative research)	77 (15)	230 (11.1)	32 (4.9)
Domain 4 (biomedical therapies)	75 (14.6)	201 (9.7)	28 (4.3)
Domain 5 (policy concerns)	54 (10.5)	166 (8)	30 (4.6)
Domain 6 (holistic therapies)	31 (6)	43 (2)	12 (1.8)
Domain 7 (ambiguous)	55 (10.7)	1181 (57.1)	515 (79.9)

## Results

### Participants

At the time of data analysis, 6157 of 7516 consented COPD PPRN participants had completed the baseline survey and 2286 participants had completed the longitudinal survey. 2711 participants that completed the baseline survey were ages 45–64 and 3320 were age 65 years and older. 7178 self-reported being diagnosed with COPD; the remaining 158 respondents were likely either caregivers to patients with COPD or individuals at risk for COPD. 515 respondents contributed open-ended responses to question one, and 2713 contributed responses to question two (2066 on the baseline survey and 647 on the longitudinal survey). Of those contributing open-ended responses to question 1, 67.3% were females with an average age of 64.7 (S.D. 11.8) years, and 2.3% affirmed Hispanic ethnicity; with respect to race, 83.9% identified as White, 4.5% Black, and remainder “mixed,” “other,” or not reported. 98% reported having a confirmed diagnosis of COPD.

### Data coding and adjudication

Before adjudication of the coding decisions of the 515 responses to open-ended question 1, researchers had achieved full coding agreement for 339 responses (66%), partial agreement for 98 responses (19%), and disagreement for 76 responses (15%) (see Table 1). After adjudication, 92% of all responses were coded into a single domain, 7.5% were coded into two domains, and 0.5% of the responses were coded into three domains. For the 2066 responses to question 2 from the baseline survey, before adjudication the researchers had achieved agreement for 1100 responses (53.2%), partial agreement for 112 responses (5.4%), and disagreed for 855 responses (41.3%). For the 647 responses to question 2 from the longitudinal survey, before adjudication the researchers had achieved agreement for 342 responses (52.8%), partial agreement for 14 responses (2.1%), and disagreement for 291 responses (45%).

### Domains, density mapping, and research topics

**Fig. 2:**
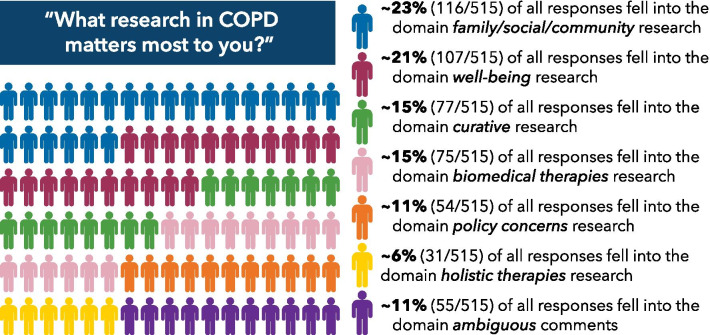
Results from categorizing participant responses to research domains

The results reported represent data captured in response to question one. After review of the data captured in response to question two, we realized the data did not provide any additional information and did not contribute to generating additional domains or research topics and therefore did not expand the results. We generated the following seven domains to capture all survey responses: 1) family/social/community research, 2) well-being, 3) curative research, 4) biomedical therapies, 5) policy concerns, 6) holistic therapies, and 7) ambiguous comments that could not be translated into research topics (see Fig. 2 and appendix for the definition of each of these domains).

#### Family/social/community research

Responses by 116 of 515 (22.5%) individuals fell into the “family/social/community research” domain. This category encompasses all responses that described research related to the connections and intimate networks of people. It included early diagnosis, genetic predispositions, education, and future prevention research. Examples of responses in this category included: “inform general public about COPD”; “looking at way I can relieve the fear and stress my family has for COPD”; “more education for medical professionals re: oxygen use”; and “determining how COPD can be prevented.” Based on all responses sorted into this domain, we distilled the following research topics:

- Identify ways to improve the care of patients in the home, including how to support COPD patients without a caregiver or someone to help them.
- Improve our understanding of genetic markers and other factors that can prevent COPD or lead to earlier diagnosis.
- Identify ways to prevent people from smoking or help them to stop smoking.
- Develop effective approaches to improve patient-provider communication.
- Develop effective educational interventions to support patients and their caregivers in dealing with COPD.
- Identify ways to improve support for families of patients with COPD.

#### Well-being

Responses by 107 of 515 (20.8%) individuals fell into the “well-being” domain. This domain encompassed all responses that reflected themes around quality of life, general physical functioning, individual patient ease and happiness, and behavioral health. Examples of responses in this category included: “best ways to stay active”; “how to live comfortably without weight on your chest”; “things to make getting around more convenient”; and “providing better quality of life whether extending life or not.” Based on all responses sorted into this domain, we developed the following research topics:

- Identify ways to reduce symptoms like cough, pain, and shortness of breath.
- Improve understanding of how long an individual may live with a COPD diagnosis.
- Identify ways to facilitate patient ease and improve quality of daily life.
- Identify ways to reduce fear, stress, anxiety, and the psychological impact of COPD.
- Identify ways to improve physical functioning and maintain mobility and independence.

#### Curative research

Responses by 77 of 515 (15%) individuals fell into the “curative research” domain. This category encompassed all responses that centered around reversing or curing the disease. Examples of responses in this category included: “to find a cure for COPD”; “stem cell treatment”; and “elimination of the disease.” Based on all responses sorted into this domain, we generated the following research topics:

- Identify ways to cure COPD, for example, with stem cells or lung regeneration.
- Identify ways to replace the diseased lung through improved or easier lung transplant.
- Identify ways to reverse COPD through new discoveries that treat the lung (other than stem-cell or transplant).

#### Biomedical therapies

Responses by 75 of 515 (14.6%) individuals fell into the “biomedical therapies” domain. This category encompassed all responses that involved traditional medication and allopathic approaches to mitigate the effects of COPD or decrease the frequency of exacerbations. This includes drug development, oxygen interventions, pulmonary rehabilitation, and personalized medicine. Examples of responses in this category included: “research regarding chronic bronchiectasis and how to reduce mucous production”; “pulmonary hypertension”; and “research to develop improved portable oxygen concentrators.” Based on all responses sorted into this domain, we generated the following research topics:

- Identify ways to improve drugs that relieve shortness of breath or treat flare-ups.
- Identify ways to improve medical equipment such as oxygen delivery systems or CPAP machines.
- Identify ways to personalize treatments using genetic or other information about individual patients.
- Identify ways to treat COPD along with other diseases patients may have, such as asthma or sleep apnea.

#### Policy concerns

Responses by 54 of 515 (10.5%) individuals fell into the “policy concerns” domain. This category encompassed all responses that described research about governmental regulations, including environmental and pollution effects. It centered around topics that affect or alter the ability to treat COPD, such as access to medical insurance, cost concerns, and policy effects on patients and the medical system. Examples of responses in this category included: “price of medication;” “environmental contributions to flare ups;” and “being able to access a lung specialist for treatment.” Based on all responses sorted into this domain, we generated the following research topics:

- Identify ways to improve insurance and drug coverage to support access to drugs and medical devices.
- Improve our understanding of how jobs, environment, and pollution cause or affect COPD.
- Improve our understanding of how governmental regulations and health policies impact the lives of those with COPD.

#### Holistic therapies

Responses by 31 of 515 (6%) individuals fell into the “holistic therapies” domain. This category encompassed all responses that focused on non-medical disease mitigation through complementary, alternative, and non-traditional approaches. Examples of responses in this category included: “medical marijuana as treatment,” “would like to get off oxygen how about vapor,” “natural remedies instead of medications.” Based on all responses sorted into this domain we generated the following research topics:

- Ways to use diet, vitamins, or nutrition to reduce the effects of COPD.
- Ways to use complementary, alternative, or other non-traditional approaches, including holistic therapies like yoga, to treat or reduce the effects of COPD.

#### Ambiguous comments

Responses by 55 of 515 (10.7%) individuals fell into the “ambiguous comments” domain. This category encompassed all responses that expressed vague constructs that could not easily be translated into concrete research topics. Examples of responses in this category included: “help I’m not sure what else can we do;” “my friends on oxygen need help;” and “really do not have any problems”.

## Discussion

In this manuscript, we present novel, patient-generated COPD research domains and topics. We established seven domains to categorize several thousand comments by COPD patients in response to the question “What research in COPD matters most to you?” We then created preliminary research topics congruent with density mapping from commonly expressed themes. This study addresses the increasingly recognized importance of ensuring that research addresses topics relevant to patient and caregiver stakeholders.

Our findings demonstrate that COPD patients and caregivers see a great need for research that supports their broader communities: nearly a quarter of all responses were categorized to this domain. Research to prevent future generations from contracting COPD and research to develop better educational materials to benefit the dependents of current COPD patients were important to COPD patients and their caregivers. Patients also frequently mentioned the need for research into raising awareness about COPD among the general public through effective outreach. Nearly a quarter of all responses also included suggestions for research that addresses the overall well-being of COPD patients, emphasizing the importance of quality of life, general physical functioning, and individual patient ease and happiness to COPD patients and their caregivers.

The research topics generated by people living with COPD differed in content from prior scientist-led efforts to develop priorities for COPD research. A discrepancy between scientist-led and patient-led efforts for developing COPD-research priorities has been noted in the past [12]. Prior COPD research-topic prioritization efforts resulted in prioritizing research around chronic care, care coordination, acute care, and transitions in care [17]. In another study, all research topics that were prioritized focused on biomedical interventions of COPD [16]. These results are markedly different from the requests of patients and their caregivers into research that investigates social and behavioral implications of living with COPD that surfaced here.

While some of the topics that emerged as important to COPD patients during this process have been the subject of extensive prior research, such as smoking prevention and cessation [18, 19], many others have not been major focuses of research on COPD to date. Common topics in COPD research include treatment of exacerbations [20, 21], the role of physical activity in disease management [22, 23], mental health concerns [24, 25] and the impact of environmental factors on disease development and progression [26, 27]. Research about the implications of living with COPD for patients, their families, and the larger communities in which they live was highlighted here as important, but to date has gained less attention in the research community [28]. Patient-identified topics also vary in their feasibility: patient and caregiver interest in research to cure COPD may be possible for some groups of patients, but is unlikely to be successful for others, depending on the type of COPD [29]. The data presented here provide key information about the breadth of patient concerns and ideas for COPD research, as well as insights into how COPD patients think about the role of COPD research. Next steps in developing this research agenda will include considerations of feasibility and relationship to ongoing research.

### Limitations

Only English-speaking patients and their family members and caregivers who have access to the internet and are registered users of the COPD PPRN website could participate in the study. This may have limited the representativeness of the sample; indeed, we found that non-Hispanic Whites and female participants who responded to the specific question about which research matters most to them were overrepresented in this sample [30]. Additionally, not everyone registering with the COPD PPRN website chose to fill in the open-ended questions that this analysis was based on. However, data was collected from over two thousand participants, a larger sample than any previous efforts to gather stakeholder input.

No people living with COPD or caregivers participated in generating the research domains or analyzed or interpreted participants’ responses. However, the COPD PPRN governing board and a broad stakeholder group did participate in the development, oversight, and interpretation of the work. The researchers who analyzed the data and generated research domains were subject-matter experts in various fields, and included staff representing the COPD Foundation, a COPD advocacy group. Next steps for this project will provide patients with an opportunity to have a direct say in shaping the research agenda. These research items will undergo vetting both with patient-collaborating investigators and with a broad stakeholder group to aid policymakers in determining COPD-related research priorities. Crowdsourced voting on COPD360Social (an online community with over 40,000 patient users) will be used to create a patient-led COPD community prioritization for a COPD patient-driven research agenda from the identified patient-generated items.

## Conclusion

Using qualitative open-ended survey responses from the COPD PPRN registrants, we were able to identify six key domains of research about COPD that are considered most important by patients. These domains differ in content from prior scientist-led efforts to develop priorities for COPD research, demonstrating the ongoing importance of involving patients and their caregivers in determining research priorities. The results suggest the field can more closely align research efforts to patient priorities by considering the identified domains.

## Data Availability

The datasets generated and/or analyzed during the current study are available from the corresponding author on reasonable request.

## Appendix: Domain definitions

1. Family—Social—Community Research: research in this domain includes educational effects to both patients and their relations, home needs, social isolation, effects on loved ones, burden, and family concern research. This research is more related to the connections and intimate networks of people than the larger societal policy domain themes or specific aspects of well-being. Early diagnosis, genetic predisposition, tobacco cessation research that is not policy nor biomedical drug focused, and future prevention would be included in this domain.
2. Well-Being: research topics in the domain of patient well-being reflect themes centered around individuals feeling better, maintaining mobility and independence, general experience with COPD as a disease by individual patients, behavioral and mental impact of disease, and palliative/end-of-life care treatments including symptoms related to COPD or exacerbations. This domain is distinct and separate from prevention, education, curative, or any specific therapies that might be researched be they alternative/complementary or biomedical in nature and is more focused on behavioral health, quality of life, general physical function, and individual patient ease/happiness.
3. Curative Research: research in this domain is centered around reversing the disease of COPD or study of interventions that might result in cure. Topics here are focused on lung regeneration, transplantation, stem cell, or other novel approaches or new discovery to not just treat symptoms or mitigate the disease experience but on eliminating COPD as a disease.
4. Biomedical Therapies: research in this domain of treatments involves traditional medication and allopathic approaches to mitigating the effects of COPD or decreasing the frequency of exacerbations that include drugs, oxygen, specific interventions like pulmonary rehabilitation, and modifying effects like genetic implications for personalized medicine; multimorbidity like co-existent asthma or other conditions are included.
5. Policy Concerns: research in this domain centers around topics that affect or alter the ability to treat COPD such as research on insurance plans, cost concerns, policy effects either on the patient or medical systems; it is separate from specific therapy or behavioral approaches. This domain is meant to encompass distributed constructs in society like research into causes, environmental/pollution effects, or health system consequences of governmental regulations.
6. Holistic Therapies: research in this domain of treatment and COPD disease mitigation center around non-medical, diet/nutrition, complimentary, alternative, and non-traditional approaches. It includes specific research modalities or approaches; not behavioral aspects that effect general well-being or experiential research and not policy/social/population work.
7. Other / Ambiguous Comments: responses that are difficult to relate to any of the above research domains or that express non-descript constructs are included in this final category; examples include “helping others”, “thank you”, “nothing at the moment”, or “I was in a drug trial.” Succinct phrases or lengthy stories that can relate to categorizable research ideas should be categorized into the other domains whenever possible unless no emergent theme is identified. The qualitative scientists will strive to examine responses and if possible, focus comments and expressed patient-caregiver ideas towards emergent researchable topics that can be rigorously studied with the goal of providing actionable data.

## Abbreviations

COPD: Chronic Obstructive Pulmonary Disease
PPRN: Patient-Powered Research Network
